# Seasonal Betacoronavirus Antibodies’ Expansion Post-BNT161b2 Vaccination Associates with Reduced SARS-CoV-2 VoC Neutralization

**DOI:** 10.1007/s10875-021-01190-5

**Published:** 2022-01-09

**Authors:** Stefania Dispinseri, Ilaria Marzinotto, Cristina Brigatti, Maria Franca Pirillo, Monica Tolazzi, Elena Bazzigaluppi, Andrea Canitano, Martina Borghi, Alessandra Gallinaro, Roberta Caccia, Riccardo Vercesi, Paul F. McKay, Fabio Ciceri, Lorenzo Piemonti, Donatella Negri, Paola Cinque, Andrea Cara, Gabriella Scarlatti, Vito Lampasona

**Affiliations:** 1 Viral Evolution and Transmission Unit, IRCCS Ospedale San Raffaele, 20132 Milan, Italy; 2 Diabetes Research Institute, IRCCS Ospedale San Raffaele, 20132 Milan, Italy; 3 National Center for Global Health, Istituto Superiore Di Sanità, 00161 Rome, Italy; 4 Department of Infectious Diseases, Istituto Superiore Di Sanità, 00161 Rome, Italy; 5 Unit of Infectious Diseases, IRCCS Ospedale San Raffaele, 20132 Milan, Italy; 6 Department of Infectious Disease, Imperial College, London, UK; 7 School of Medicine and Surgery, Università Vita-Salute San Raffaele, 20132 Milan, Italy; 8 Hematology and Bone Marrow Transplantation Unit, IRCCS Ospedale San Raffaele, 20132 Milan, Italy

**Keywords:** COVID-19, Vaccine, Antibodies, Neutralizing antibodies

## Abstract

SARS-CoV-2 vaccination is known to induce antibodies that recognize also variants of concerns (VoCs) of the virus. However, epidemiological and laboratory evidences indicate that these antibodies have a reduced neutralization ability against VoCs. We studied binding and neutralizing antibodies against the Spike protein domains and subunits of the Wuhan-Hu-1 virus and its alpha, beta, delta VoCs and of seasonal betacoronaviruses (HKU1 and OC43) in a cohort of 31 health care workers prospectively followed post-vaccination with BNT162b2-Comirnaty. The study of sequential samples collected up to 64 days post-vaccination showed that serological assays measuring IgG against Wuhan-Hu-1 antigens were a poor proxy for VoC neutralization. In addition, in subjects who had asymptomatic or mild COVID-19 prior to vaccination, the loss of nAbs following disease could be rapid and accompanied by post-vaccination antibody levels similar to those of naïve vaccinees. Interestingly, in health care workers naïve for SARS-CoV-2 infection, vaccination induced a rapid and transient reactivation of pre-existing seasonal coronaviruses IgG responses that was associated with a subsequent reduced ability to neutralize alpha and beta VoCs.

**Supplementary Information:**

The online version contains supplementary material available at 10.1007/s10875-021-01190-5.

## Introduction

Large-scale SARS-CoV-2 vaccination campaigns are ongoing although with large disparities between countries [1]. While antibodies to SARS-CoV-2 are not the only determinant of immunity, they are important predictors of immunological protection from SARS-CoV-2 infection or disease [2]. However, the continuous worldwide emergence of SARS-CoV-2 variants of concern (VoC) is challenging the effectiveness of licensed vaccines with several open questions remaining about the effectiveness and persistence of vaccine-induced antibodies [3, 4]. Public health policies need to adapt on a rolling basis and several governments are already introducing additional rounds of vaccination in subjects at risk of severe disease [5]. Moreover, the contribution to community immunity of subjects who experienced an asymptomatic or mild COVID-19 is unclear.

To address some of these issues, in this study, we analyzed prospectively the antibody response to SARS-CoV-2 and seasonal betacoronaviruses induced by BNT162b2-Comirnaty vaccination in a cohort of health care workers (HCW) with or without a prior COVID-19. Overall, our results suggest that (1) vaccine-induced Wuhan-Hu-1 RBD IgGs should be used with caution as proxy for VoC neutralization; (2) in subjects who had asymptomatic or mild COVID-19, the loss of neutralizing antibodies (nAbs) following disease can be rapid and accompanied by antibody levels post-vaccination no greater than in naïve vaccinees, suggesting that protection from re-infection mediated by antibodies would be no better than in naïve subjects; and (3) we observed the reactivation of pre-existing seasonal coronaviruses’ antibody responses following BNT162b2-Comirnaty vaccination.

## Methods

### Study Approval

All procedures involving human participants were in accordance with the ethical standards of the institutional and/or national research committee and with the 1964 Helsinki declaration and its later amendments or comparable ethical standards. Written informed consent was obtained from all individual participants included in the study.

The study subjects belonged to the IRCCS Ospedale San Raffaele COVID-19 cohort study COVID-BioB (registered as ClinicalTrialsgov-NCT04318366) approved by the IRCCS Ospedale San Raffaele Ethics Review Board (protocol 68/INT/2020) and to the COVID-BioB related immunological sub-study “Role of the immune response in the infection with SARS-CoV-2 and in the pathogenesis of COVID-19” (protocol number 34/INT/2020).

### Study Population

The study subjects included 31 hospital health care workers (HCW) of the IRCCS Ospedale San Raffaele, who received their first dose of the Pfizer-BioNtech mRNA vaccine BNT162b2-Comirnaty between January 7th and March 5th, 2021. All but two subjects received their second vaccination jab after 21 days according to Italian national guidelines (https://www.gazzettaufficiale.it/atto/serie_generale/caricaDettaglioAtto/originario?atto.dataPubblicazioneGazzetta=2021-03-24&atto.codiceRedazionale=21A01802&elenco30giorni=false) (Supplemental Table S1). Thirteen were naïve to COVID-19 at vaccination, while 18 had a previous SARS-CoV-2 infection either during the first pandemic wave, between February and March 2020 (*n* = 13, median interval from symptoms onset to first jab 302.5 days), or during the second wave, between October and November 2020 (*n* = 5, median interval from symptoms onset to first jab 76 days). Four individuals with COVID-19 with a confirmed negative nasopharyngeal swab post-infection tested positive at a routine in-hospital screen 1.5 to 8 months later likely due to asymptomatic re-infection events.

A confirmed infection case was defined as a SARS-CoV-2-positive real-time reverse-transcriptase polymerase chain reaction (RT-PCR) from a nasopharyngeal swab and/or COVID-19 symptoms and/or a serologic evidence of antibody to SARS-CoV-2. The WHO classification was used to define disease severity (https://www.who.int/publications-detail-redirect/WHO-2019-nCoV-clinical-2021-1). Asymptomatic infections were captured thanks to the hospital SARS-CoV-2 testing policy, which introduced in May 2020 a serologic antibody test (LIAISON® SARS-CoV-2 S1/S2 IgG serological test, DiaSorin S.p.A., Vercelli, Italy) and in August 2020 a regular monthly or bimonthly SARS-CoV-2 nasal-pharyngeal swab test. The clinical and laboratory data were collected from medical chart review or directly by interview, crosschecked for accuracy by data managers and clinicians, and entered in a dedicated electronic case record form (eCRF) developed on site for the COVID-BioB study in MySQL (V5.7.14) on Apache Tomcat (V2.4.23) platform running on windows sever 2012 R2.

Biological material of the vaccinees included a dedicated serum sample collected at first vaccination and thereafter at 10, 21, 31, 42, and 64 days (with an approximation of ± 5 days). All serum samples were coded and anonymously processed for the immunological studies.

### Construction of Plasmids for Lentiviral Pseudotypes’ Expression of Variant Spikes

A schematic representation of recombinant antigens used in this study is shown in Supplemental Figure S1. Briefly, the plasmid pSpike-C3 encodes a codon optimized SARS-CoV-2 Spike protein open reading frame (ORF) (GenBank: NC_045512.2) containing a 21 amino acid deletion at the cytoplasmic tail (delta21) of Spike protein as previously described [6]. For construction of pSpike-UKC3 and pSpike-SAC3 plasmids, expressing the variant Spike ORFs with a 21 amino acid deletion at the cytoplasmic tail, a NheI/AvaI fragment of DNA was removed from Alpha (B.1.1.7 pSpike-UK) and Beta (B.1.351 pSpike-SA) plasmids and inserted into the corresponding restriction sites of pSpike-C3 plasmid, to obtain pSpike-UKC3 and pSpike-SAC3 plasmids. Plasmid pSpike-INd19 was constructed by inserting a codon optimized B.1.617.2 Spike (Delta) sequence with a 19 amino acid deletion at the cytoplasmic tail into HindII/NotI restriction sites of pcDNA3.1 expression plasmid. The B.1.1.7 Alpha pSpike-UKC3 used in these studies contained the following mutations: del69-70HV, del145Y, N501Y, A570D, D614G, P681H, T716I, S982A, D1118H. The B.1.351 Beta pSpike-SAC3 used in these studies contained the following mutations: L18F, D80A, D215G, del242-244LAL, R246I, K417N, E484K, N501Y, D614G, A701V. The B.1.617.2 Delta pSpike-INd19 used in these studies contained the following mutations: T19R, del157-158, L452R, T478K, D614G, P681R, D950N (Supplemental Fig. S1).

### Production of a Simian Lentiviral Vector Expressing Luciferase (LV-Luc) and Pseudotyped with Spike Variants

293 T Lenti-X human embryonic kidney cell line (Clontech, Mountain View, CA, USA) was used for production of LV-Luc pseudotyped with Spike variants by transient transfection as previously described [6]. Briefly, 293 T Lenti-X cells (3.5 × 106 cells) were seeded on 10-cm Petri dishes (Corning Incorporated—Life Sciences, Oneonta, NY, USA) and transiently transfected with plasmid pGAE-LucW, pADSIV3 + and pseudotyping plasmid (pSpike-C3, pSpike-UKC3, pSpike-SAC3, pSpkeINd19) using the JetPrime transfection kit (Polyplus Transfection Illkirch, France) following the manufacturer’s recommendations using a 1:2:1 ratio (transfer vector:packaging plasmid:Spike plasmid). At 48 h post-transfections, culture supernatants containing the LV-Luc pseudotypes (LV-Luc/Spike-C3, LV-Luc/Spike-UKC3, LV-Luc/Spike-SAC3, and LV-Luc/Spike-INd19) were collected and stored in 1-mL aliquots at − 80 °C until use.

### Lentiviral Vector-Based SARS-CoV-2 Neutralization Assay

LV-Luc preparations were titered on VeroE6 cells (African green monkeys, epithelial kidney). Dilutions providing 150,000–200,000 relative luciferase units (RLU) were used in the neutralization assay, as previously described [6]. Briefly, heat-inactivated serum serial threefold dilutions starting from the 1/40 dilution were incubated in duplicate with the LV-Luc for 30 min at 37 °C in 96-well plates, and thereafter added to VeroE6 cells at a density of 20,000 cells/well. After 48 h, luciferase expression was determined with a luciferase assay system (Bright-Glo, Promega) and measured in a Mitras luminometer (Berthold, Germany). The 50% inhibitory serum dilution (ID50) was calculated with a linear interpolation method using the mean of the duplicates [7]. Neutralization was expressed as the reciprocal of the serum dilution giving 50% inhibition of RLU compared to the mean of the virus control wells. An ID50 below 1/40 serum dilution was considered negative and a value of 10 ascribed for statistical analysis.

### IgG Binding Antibody Luciferase Immunoprecipitation System (LIPS) Assay

Using LIPS [8], we measured IgG binding to recombinant nanoluciferase tagged antigens corresponding to the following: SARS-CoV-2 Wuhan-Hu-1, alpha, beta, and delta spike RBD domains; Wuhan-Hu-1 spike S2 subunit; HKU1 and OC43 seasonal betacoronaviruses’ spike S1 B domain and spike S2 subunits; pandemic Ca2009 H1N1 influenza virus hemagglutinin (HA) antigen, as previously described [9, 10] (Supplemental Figure S1). Viral sequences used in this study correspond to the following deposited sequences: GenBank NC_045512.2 for SARS-CoV-2 Wuhan, GenBank AGW27863.1 for HKU1 [11], MZ450972.1 for OC43. HKU1 and OC43 viral sequences correspond to isolates reported multiple times in the USA, China, and Japan over the last decade. Briefly, we cloned recombinant nanoluciferase-tagged monomeric or multimeric antigens and expressed them by transient transfection into Expi293F™ cells (Expi293™ Expression System, Thermo Fisher Scientific Life Technologies, Carlsbad, CA, USA). For LIPS, we incubated in liquid phase each antigen with test serum (1ul) for 2 h and then captured immune-complexes with rProtein A-sepharose. After washing (5 times) the sepharose pellets, we quantified bound IgG by measuring the recovered luciferase activity in a Berthold Centro XS3 luminometer (Berthold Technologies GmbH & Co. KG, Bad Wildbad, Germany) using the MikroWin version 5.22 software. We then converted raw data into arbitrary units (AU); for SARS-COV-2-specific antibodies, we used a local positive index serum that exhibited closely similar binding to each of the VoCs, and for HKU1- and OC43-specific antibodies, we used two local index sera with strong antibody binding to the respective betacoronavirus antigen.

### Statistics

We performed statistical analyses using the R software version 3.4.1 [12]. We reported the median with either 95%CI, range, or interquartile range (IQR) for continuous variables and frequency or percent % frequency for categorical variables. We used repeated measures ANOVA to compare continuous variables over time after log transformation. Two-tailed *p* values are reported, with *p* value < 0.05 indicating statistical significance.

## Results

We used validated lentiviral vector-based SARS-CoV-2 neutralization and LIPS assays [6, 9] to profile the antibody response to spike antigens of SARS-CoV-2 (Wuhan-Hu-1, alpha, beta, and delta VoCs) and of seasonal betacoronaviruses (HKU1 and OC43) post-BNT162b2-Comirnaty vaccination (Supplemental Figure S1). We tested sera from 31 vaccinated HCW of the IRCCS Ospedale San Raffaele, Milan, Italy, with (*n* = 18) or without (*n* = 13) prior confirmed COVID-19 (Supplemental Table S1). All but two subjects received two vaccine doses 21 days apart, between January 7th and March 5th, 2021. We collected serum samples prospectively at first vaccination (baseline), and thereafter at 10, 21, 31, 42, and 64 days.

### Binding and Neutralizing Antibodies Against Wuhan-Hu-1 Antigens in BNT162b2 Vaccinees Stratified by SARS-CoV-2 Antibody Status at Baseline

At baseline before vaccination, SARS-CoV-2 Wuhan-Hu-1 nAbs and IgGs binding to spike RBD were absent in 6 out of 18 HCW with a prior history of COVID-19 and in all those without. All vaccinees responded to BNT162b2-Comirnaty immunization by producing antibodies able to both neutralize SARS-CoV-2 Wuhan-Hu-1 and bind its RBD and S2 domains (Supplemental Figure S2, A–C). Antibody binding to the Wuhan-Hu-1 spike RBD and S2 domains and neutralization titer increased above assay thresholds starting from day 10 post-vaccination. We observed a peak of antibody responses already at day 10 in HCW with prior COVID-19 and detectable SARS-CoV-2 antibodies at baseline. In HCW naïve for COVID-19 or without SARS-CoV-2 antibodies at baseline, the antibody response peaked instead at day 31, i.e., 10 days after the booster jab. Peak values were higher in HCW who presented with SARS-CoV-2 antibodies at baseline for both nAbs (ID50 geometric mean titer to Wuhan-Hu-1 at day 31: in baseline antibody positive HCW = 13,613; in baseline antibody negative HCW = 1607) and IgG to RBD and S2 spike domains (Supplemental Table S2).

### Post-vaccination, the Antibody Response Shows Significant Differences in nAb Titer but Not in IgG Binding to SARS-CoV-2 VoCs

**Fig. 1:**
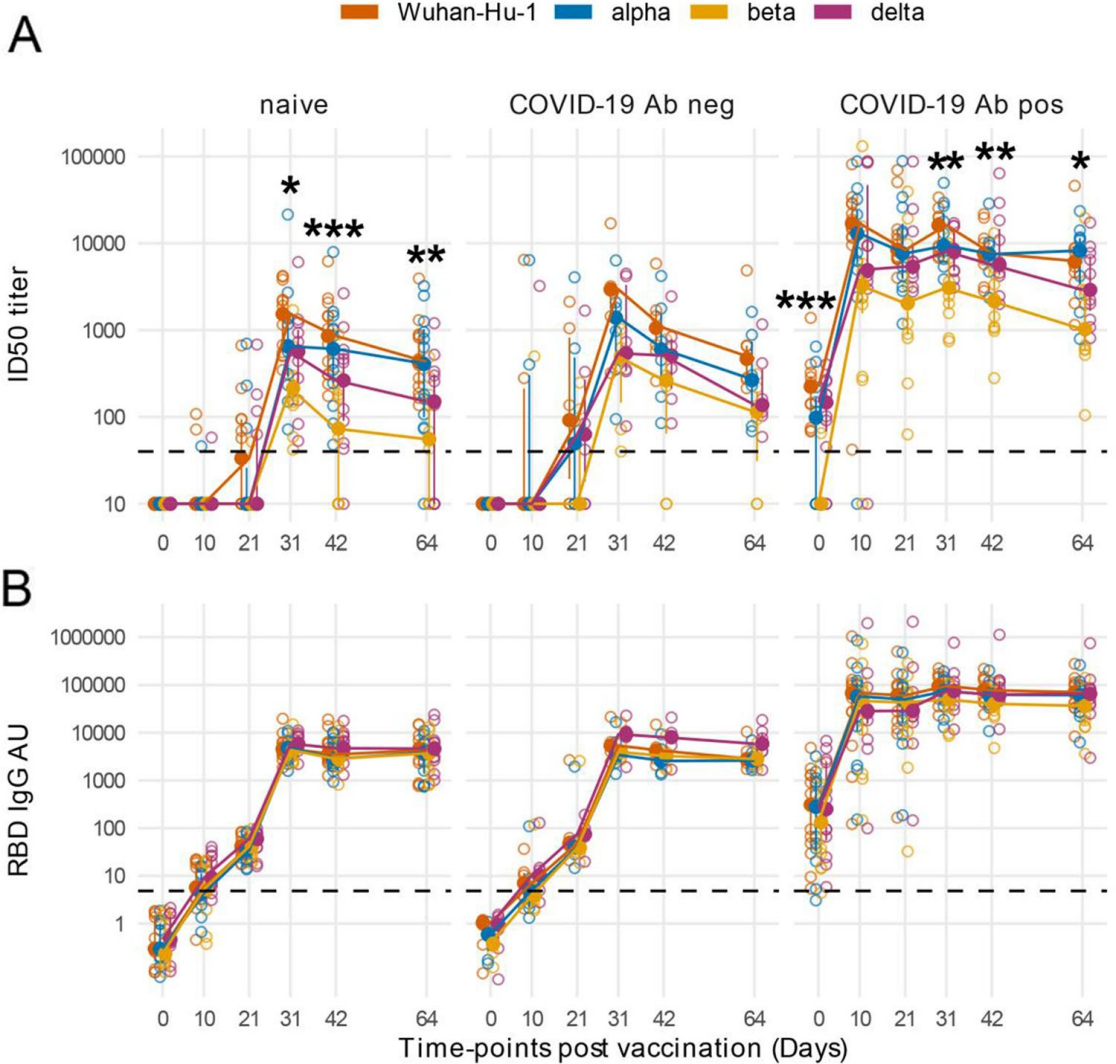
Antibody responses to Spike antigens of SARS-CoV-2 variants post-BNT162b2 vaccination show significant differences in nAbs titer but not in IgG binding to the RBD of VoCs. Line plots of ID50 titer (**A**) and RBD IgG arbitrary units (AU) (**B**) against SARS-CoV-2 Wuhan-Hu-1 and VoCs in sequential samples after vaccination. Vaccinees are presented in left to right panels stratified as follows: (left panel) subjects naïve for SARS-CoV-2 infection (*n* = 13), (middle panel) with prior confirmed COVID-19 presenting at vaccination either without Wuhan-Hu-1 nAbs and RBD IgGs (*n* = 6), and (right panel) with prior COVID-19 and SARS-CoV-2 antibodies at baseline (*n* = 12). Filled circles and bars represent the median ± inter quartile range at each time-point, and empty circles show each individual measurement. The horizontal dashed lines stand for the threshold for positivity. The asterisks indicate statistically significant differences in mean titers of nAbs or RBD IgGs levels across VoCs at the corresponding time-points (two-way repeated measures ANOVA, *p adjusted < 0.05, **p adjusted < 0.01, ***p adjusted < 0.001)

NAbs and RBD IgGs against SARS-CoV-2 alpha, beta, and delta VoCs had post-vaccination kinetics similar to that against Wuhan-Hu-1, with delayed and lower peak titers in COVID-19 vaccinees without detectable antibodies at baseline (Fig. 1A, and B). NAb peak titers against VoCs were reduced compared with nAbs to Wuhan-Hu-1. The reduction of peak VoC beta nAb titer was statistically significant both in HCW naïve for COVID-19 or who presented with SARS-CoV-2 antibodies at baseline (Fig. 1A and Supplemental Table S2).

NAbs declined already from day 42 after the vaccination in HCW without nAbs at baseline (day 64/day 31 nAb geometric mean titer ratio in HCW naïve: 0.37, 0.43, 0.23, and 0.19; in HCW nAb negative: 0.23, 0.30, 0.22, and 0.27, for Wuhan-Hu-1, alpha, beta, and delta respectively) (Supplemental Table S2). At the end of follow-up, nAbs dropped below the threshold for positivity in 7 HCW against the VoC beta (all of whom were antibody negative at baseline: 5 naïve for SARS-CoV-2 infection and 2 with prior COVID-19) and in 4 against the VoC delta (all naïve for SARS-CoV-2 infection). Conversely, IgG binding to Wuhan-Hu-1 and VoC RBD domains was comparable and showed only a modest drop by day 64 (Fig. 1B).

### Post-vaccination, Antibody Levels to Spike Antigens of SARS-CoV-2 and Its Variants Are Correlated to Previous Disease Severity

**Fig. 2:**
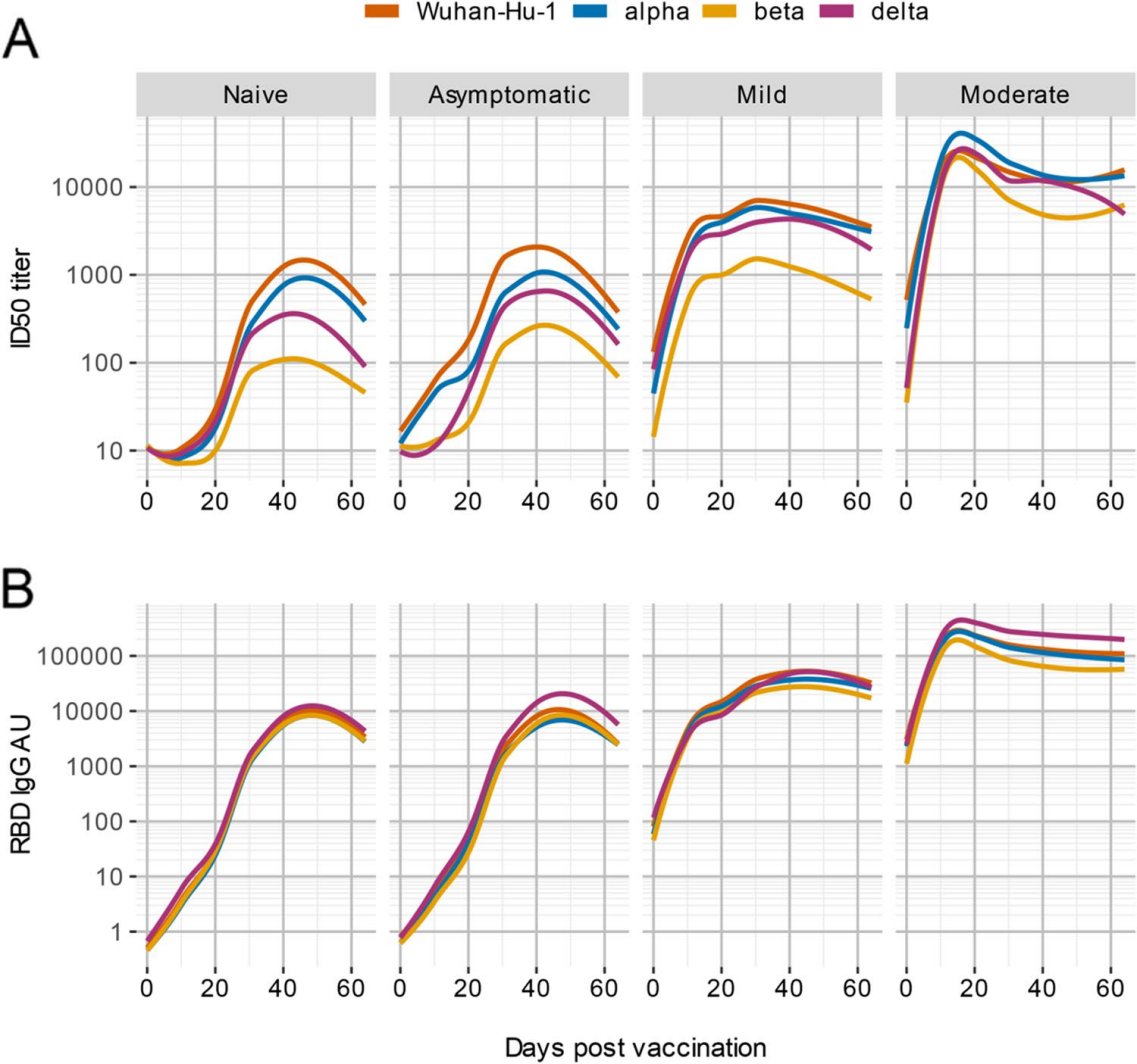
Post-BNT162b2 vaccination, antibody levels to spike antigens of SARS-CoV-2 and its variants are correlated to previous disease severity. The line plots show the moving average of antibody levels against the indicated SARS-CoV-2 variants after fitting of a LOESS polynomial regression. Subjects are stratified according to symptoms after SARS-CoV-2 infection. Antibody responses to Spike antigens of SARS-CoV-2 VoCs post-BNT162b2 vaccination show differences in nAbs titer (**A**) but not in IgG binding to the RBD (**B**)

HCW with prior COVID-19 were stratified according to disease severity: disease was asymptomatic in 5, mild in 11, and moderate in 2 (Supplemental Table S1). After BNT162b2 vaccination, we observed that nAb peak values increased according to disease severity in HCW (Fig. 2A). The reduction of nAb titer against VoCs was particularly evident in naïve subjects or who previously had asymptomatic or mild symptoms. RBD IgG arbitrary units also showed a correlation with previous disease severity but the difference in peak IgG binding between Wuhan-Hu-1 spike and VoCs antigens within each HCW category was not significant (Fig. 2B).

### An Early Boost of HKU1 IgGs Post-vaccination Is Associated with a Rapidly Decreasing Ab Response Against SARS-CoV-2 Antigens in COVID-19-Naïve Subjects

**Fig. 3:**
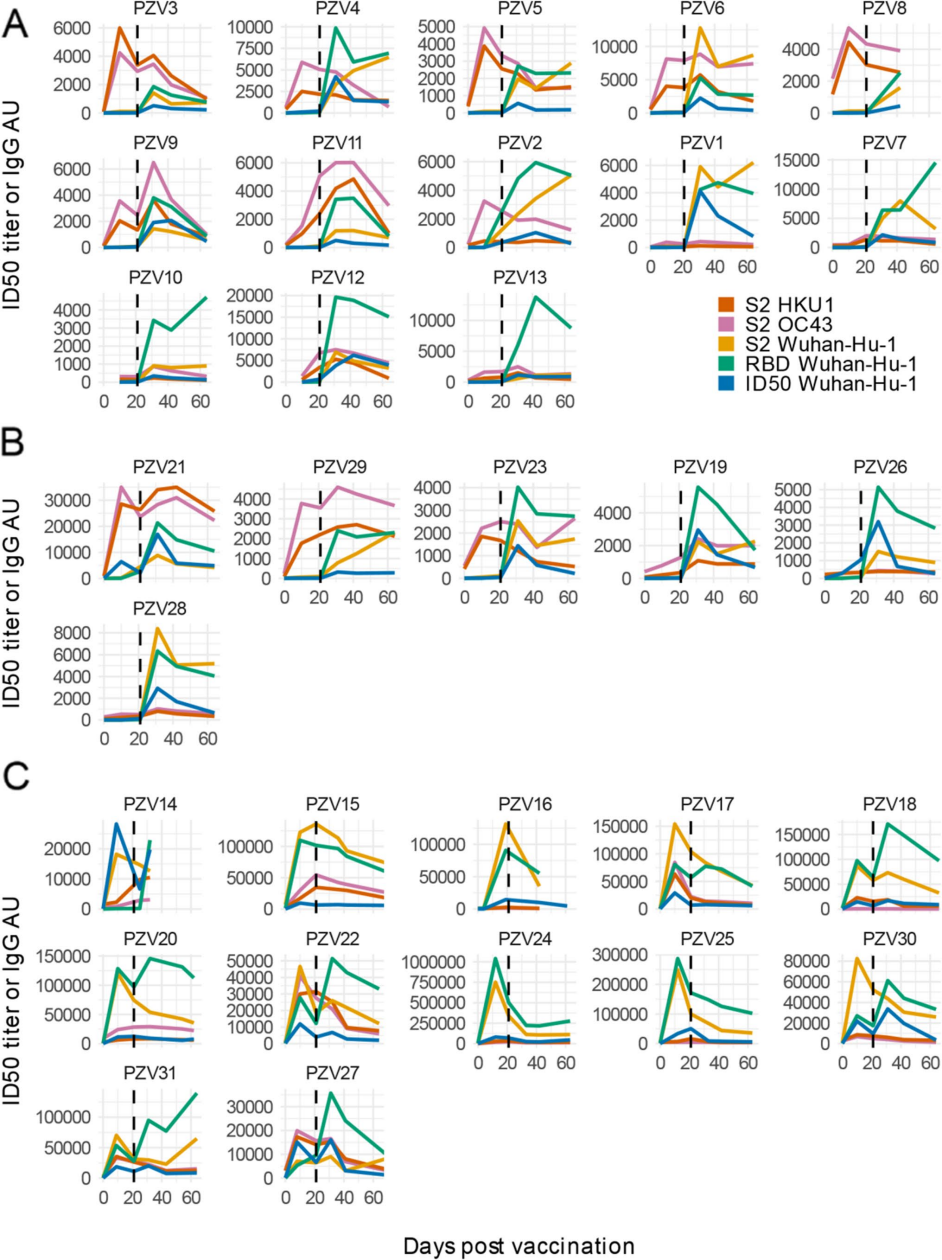
BNT162b2-Comirnaty vaccination can induce a large, early boost of antibodies against seasonal betacoronaviruses in COVID-19-naïve subjects. Line plots of ID50 nAbs and RBD IgG arbitrary units (AU) to SARS-CoV-2 Wuhan-Hu-1 and S2 spike proteins of SARS-CoV-2, OC43, and HKU1 betacoronaviruses at sequential time-points after vaccination. Vaccinees are stratified as follows: (**A**) subjects naïve for SARS-CoV-2 infection (*n* = 13), (**B**) subjects with prior confirmed COVID-19 presenting at vaccination either without Wuhan-Hu-1 nAbs and RBD IgGs (*n* = 6), and (**C**) with prior COVID-19 and SARS-CoV-2 antibodies at baseline (*n* = 12). The vertical dashed line indicates the 2nd vaccine jab

Vaccination induced a large, early boost of IgG against the extracellular domain of the OC43 and HKU1 seasonal betacoronaviruses’ Spike S2 subunits in a fraction of HCW. The rise of seasonal coronaviruses Spike S2 antibodies occurred already at day 10 after the first vaccination in 7 out of 13 naïve HCW and 3 of 6 HCW with prior COVID-19 (all SARS-CoV-2 Abs negative at baseline) (Fig. 3A, and B). In all HCW who presented with SARS-CoV-2 Abs at baseline, the Ab response against Wuhan-Hu-1 spike antigens at day 10 invariably exceeded that against seasonal betacoronaviruses S2 (Fig. 3C). The expanded IgG response to HKU1 and OC43 S2 antigens appeared to be minimally if at all cross-reactive with SARS-CoV-2 S2. No increase in IgG binding was instead observed against the Spike S1 B domain region of both seasonal betacoronaviruses that corresponds to the RBD on SARS-CoV-2 (Supplementary Figures S3–S4). This was consistent with the greater aminoacid sequence homology between seasonal beta coronaviruses and SARS-CoV-2 Spike S2 extracellular domains compared to the Spike S1 B and RBD domains (Supplementary Figures S5–S6).

To exclude a generalized impact of vaccination on pre-existing antibody responses, we measured IgG binding to the HA antigen of the 2009 pandemic H1N1 flu virus in COVID-19-naïve HCW. HA antibodies showed modest fluctuations during follow-up, which were not synchronous with those against betacoronaviruses antigens (Supplemental Figure S7).

**Fig. 4:**
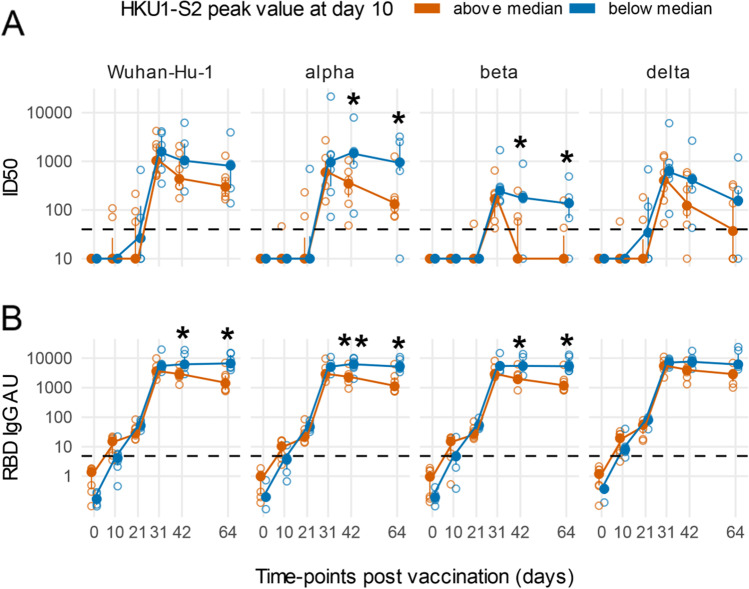
An early boost of HKU1 IgGs post-vaccination is associated with a rapidly decreasing Ab response against SARS-CoV-2 in COVID-19-naïve subjects. Line plots of Wuhan-Hu-1 and indicated VoCs ID50 nAbs (**A**) and RBD IgG arbitrary units (AU) (**B**). Subjects naïve for COVID-19 were stratified as above or below the median of HKU1 S2 IgGs at day 10 post-vaccination. Filled circles and bars represent the median ± inter quartile range at each time-point, and empty circles show each individual measurement. The horizontal dashed lines stand for the threshold for positivity. The asterisks indicate statistically significant differences in mean ID50 nAbs or RBD IgG AU at the corresponding time-points between subjects with or without an early boost of seasonal coronaviruses responses after vaccination (two-way repeated measures ANOVA, *p < 0.05, **p < 0.01)

In SARS-COV-2-naïve HCW, HKU1 S2 IgGs above the median levels at day 10 post-vaccination were associated with a lower peak and a faster decline during follow-up of SARS-CoV-2 nAbs against Wuhan-Hu-1, alpha, and beta variants (two-way repeated measures ANOVA *p* adjusted < 0.015) (Fig. 4A). A similar trend was observed also for nAbs against the delta variant but did not reach statistical significance. At day 64 post-vaccination, in the 7 naïve subjects with an early boost of HKU1 antibodies, nAbs dropped below detection limit in 4 against the beta VoC and in 3 against the delta VoC, compared to 1 of 6 of those without the early boost of seasonal betacoronaviruses antibodies. A similar but statistically not significant trend was present also in HCW with prior COVID-19 who presented at vaccination without SARS-CoV-2 antibodies (Supplemental Figure S8A). No differences in nAbs against the variants were present in HCW who at baseline had detectable SARS-CoV-2 antibodies (Supplemental Figure S9A).

Similarly, always in HCW COVID-19 naïve with an early boost of HKU1 antibodies, we observed during follow-up a modest but significant peak reduction and decline of IgG against the SARS-CoV-2 RBD that reached statistical significance for Wuhan-Hu-1, alpha, and beta variants (two-way repeated measures ANOVA *p* adjusted < 0.05) (Fig. 4B). Stratification according to HKU1 IgG boost of HCW with prior COVID-19 was not associated with significant differences in SARS-CoV-2 RBD IgGs during follow-up (Supplementary Figures S8B–S9B).

## Discussion

The results of our study indicate that vaccinees naïve for COVID-19 frequently show a rapid drop below detection limit of antibodies that cross-neutralize VoCs in vitro. Interestingly, a similar profile of post-vaccination antibodies was seen in some vaccinees who recovered from prior asymptomatic or mild COVID-19 and were Wuhan-Hu-1 nAbs negative at the time of vaccination. This was at variance with the outcome in vaccinees with prior COVID-19 with detectable Wuhan-Hu-1 nAbs at baseline, who had a neutralizing response an order of magnitude greater and sustained throughout follow-up. While the results of our in vitro test do not entirely recapitulate the ability of the vaccinees’ immune system to stave off SARS-CoV-2 infection, this rapid decrease of antibodies able to neutralize VoCs is consistent both with the recently reported drop of vaccine effectiveness after the delta variant began to widely circulate and increased viral load in vaccinated individuals infected by VoCs [13].

Moreover, our study shows that the nAb response kinetics is not closely mirrored by that of IgG binding to the RBD. IgG binding to the Wuhan-Hu-1 and VoC RBDs was similar and showed only a modest drop by the end of the follow-up. This observation agrees with the recent evidence that many RBD IgGs in vaccinees are non-neutralizing and that several nAbs target the spike protein NTD rather than the RBD [14]. Therefore, caution should be exercised in using RBD antibodies as proxy for VoC neutralization or as a surrogate marker to predict vaccine effectiveness. Essentially all current large-scale screening immunoassays that measure RBD antibodies are based on the Wuhan-Hu-1 sequence. Our study suggests that simply switching the RBD antigen sequence to that of VoCs would not lead to an efficient detection of differences in neutralizing activity among screened sera.

Furthermore, our data shows that BNT162b2 vaccination frequently induces an early boost of antibodies to the S2 subunit of OC43 and HKU1 seasonal betacoronaviruses. This boost was rapid and large in > 50% of vaccinees naïve for SARS-CoV-2 infection. Interestingly, the rapid increase of IgG to HKU1 at 10 days post-mRNA vaccination was associated with a trend to faster decrease and subsequent loss of SARS-CoV-2-specific antibodies. While a confirmation in a larger cohort is warranted in light of the limited number of subjects analyzed in this study, in naïve vaccinees, a strong reactivation of pre-existing seasonal coronaviruses antibody responses was associated with a statistically significant subsequent reduction of nAbs against the alpha and beta VoCs. In the case of the beta VoC, which is currently thought as the most immune evasive [15], this was also associated with a rapid loss of neutralization.

We previously described an increase of antibodies against HKU1 and OC43 Spike antigens in a cohort of symptomatic COVID-19 patients shortly after disease onset [9]. In those patients, HKU1 and OC43 antibody levels were significantly correlated with those of SARS-CoV-2-specific antibodies during the first 3–4 weeks from symptoms onset. The existence of cross-reactive antibodies between SARS-CoV-2 and seasonal betacoronaviruses has been demonstrated also by other studies [16, 17] and further supported by the isolation of cross-reactive monoclonal antibodies from germinal centers of vaccinated individuals [14]. The coding sequence of these cross-reactive monoclonal had more extensive somatic mutations compared to non-cross-reactive ones, signaling a likely derivation from memory responses pre-existing vaccination. Moreover, these findings are consistent with the existence of betacoronaviruses cross-reactive T cells in subjects that were either naïve for SARS-CoV-2 infection or had COVID-19 [2, 18, 19].

It is relatively straightforward to speculate that the observed early rapid expansion of HKU1 and OC43 IgG antibodies post-vaccination could be attributed to the presence of both elevated pre-existing memory B cells to seasonal coronaviruses and cross-reactive helper T cells recognizing peptide epitopes in the Spike S2 subunit, due to its relatively high homology between coronaviruses. However, it is unclear how the expansion of memory responses to other betacoronaviruses Spike S2 might subsequently impact on SARS-CoV-2 nAbs development. The S2 subunit is rarely if ever directly targeted by SARS-CoV-2 nAbs [20]. It is therefore unlikely that an early deviation of antibody responses toward the S2 of seasonal betacoronaviruses rather than that of SARS-CoV-2 might lead to a direct decrease of nAbs. Furthermore, our data suggest also a partially generalized reduction of SARS-CoV-2-specific antibodies including binding IgG against the spike RBD in naïve subjects with an early reactivation of seasonal betacoronaviruses’ B cell memory. Possibly, this observation might support the concept of a preferential and synchronous expansion of a subset of pre-existing and cross-reactive T cells recognizing S2 rather than S1 epitopes.

The persistence of nAbs is key for protection from SARS-CoV-2 infection. However, modeling studies based on immunological data from mild and moderate COVID-19 patients suggest that nAbs elicited from less severe natural infection is unlikely to provide long-term protection from infection [21]. Our own data suggests that the re-expansion of pre-existing humoral memory to seasonal betacoronaviruses might dampen the SARS-CoV-2 neutralizing response induced by vaccination. Potentially, this would imply that in a relevant fraction of vaccinees naïve for COVID-19, the duration of protection from subsequent re-infection would be shortened. Additionally, it might be speculated that this mechanism might be at play also in individuals who developed SARS-CoV-2 antibodies upon infection, a fact that might contribute to the waning of SARS-CoV-2 nAbs that we observed in our study in some individuals who had an asymptomatic COVID-19 before vaccination.

While the effectiveness of the immunization with current mRNA vaccines is undisputable in reducing both infection and disease severity [22], public health policies must adapt to rapidly emerging VoCs. The choice of diagnostic tests, vaccine formulations, and vaccine deployment will benefit from investigating further the mechanisms influencing the response to vaccination, particularly in view of the ongoing development of pan-coronavirus vaccines [23].

## Supplementary Information

Below is the link to the electronic supplementary material.

Supplementary file1 (PDF 2064 KB)

## Data Availability

The data generated in the study might be made available upon reasonable request.
