# Supplementary material for: Seasonal Betacoronavirus Antibodies’ Expansion Post-BNT161b2 Vaccination Associates with Reduced SARS-CoV-2 VoC Neutralization
Source: J Clin Immunol. 2022 Jan 9;42(3):448–58. doi: 10.1007/s10875-021-01190-5 (PMC8742681; doi:10.1007/s10875-021-01190-5)
Supplement: Supplementary file 1 — Supplementary file1 (PDF 2064 KB) [file 10875_2021_1190_MOESM1_ESM.pdf]

Supplemental Table S1

Demographics and clinical characteristics of study subjects

Subject ID	Sex	Age at vaccination (years)	N. of vaccine shots	COVID-19	Symptoms category at first COVID-19	Symptoms category at second COVID-19	Days from symptoms onset to first vaccination (range)	SARS-CoV-2 alpha nAbs and RBD IgG at baseline
PZV1	F	56-60	2	Naïve				No
PZV2	M	61-65	2	Naïve				No
PZV3	F	26-30	2	Naïve				No
PZV4	F	26-30	2	Naïve				No
PZV5	M	31-35	2	Naïve				No
PZV6	M	31-35	2	Naïve				No
PZV7	F	56-60	2	Naïve				No
PZV8	F	56-60	2	Naïve				No
PZV9	F	36-40	2	Naïve				No
PZV10	F	41-45	2	Naïve				No
PZV11	M	31-35	2	Naïve				No
PZV12	F	46-50	2	Naïve				No
PZV13	M	36-40	2	Naïve				No
PZV14	F	26-30	2	First wave	asymptomatic		unknown	Yes
PZV15	F	56-60	1	First wave	moderate		350 -400	Yes
PZV16	F	46-50	1	First wave	mild		250-300	Yes
PZV17	F	41-45	2	First wave	mild		300-350	Yes
PZV18	F	51-55	2	First wave	mild		250-300	Yes
PZV19	F	51-55	2	First wave	asymptomatic		300-350	No
PZV20	M	31-35	2	First wave	mild		300-350	Yes
PZV21	F	66-70	2	First wave	mild		300-350	No
PZV22	F	56-60	2	First wave	mild		300-350	Yes
PZV23	F	46-50	2	First wave	asymptomatic		>250	No
PZV24	F	51-55	2	First wave – Second wave	moderate	asymptomatic	300-350	Yes
PZV25	F	46-50	2	First wave – Second wave	mild	asymptomatic	250-300	Yes
PZV26	M	31-35	2	First wave – Second wave	asymptomatic	asymptomatic	>200	No
PZV27	F	46-50	2	Second wave - 2020	mild	mild	50-100	Yes
PZV28	F	51-55	2	Second wave	asymptomatic		50-100	No
PZV29	F	26-30	2	Second wave	mild		50-100	No
PZV30	M	61-65	2	Second wave	mild		50-100	Yes
PZV31	F	31-35	2	Second wave	mild		50-100	Yes

Supplemental Table S2

ID50 geometric mean titer post BNT162b2 vaccination

	HCW naïve				HCW with prior COVID-19 nAbs negative at baseline				HCW with prior COVID-19 nAbs positive at baseline			
	**Wuhan-Hu-1**	**alpha**	**beta**	**delta**	**Wuhan-Hu-1**	**alpha**	**beta**	**delta**	**Wuhan-Hu-1**	**alpha**	**beta**	**delta**
**Day 31 post Vax**	1301	732	210	487	2448	959	305	708	13114	12582	3688	7609
**Day 64 post Vax**	482	312	48	94	571	285	66	195	5723	6072	1268	3310
**Day 64/31 ratio**	0.37	0.43	0.23	0.19	0.23	0.3	0.22	0.27	0.43	0.48	0.34	0.43

Supplemental figure S1

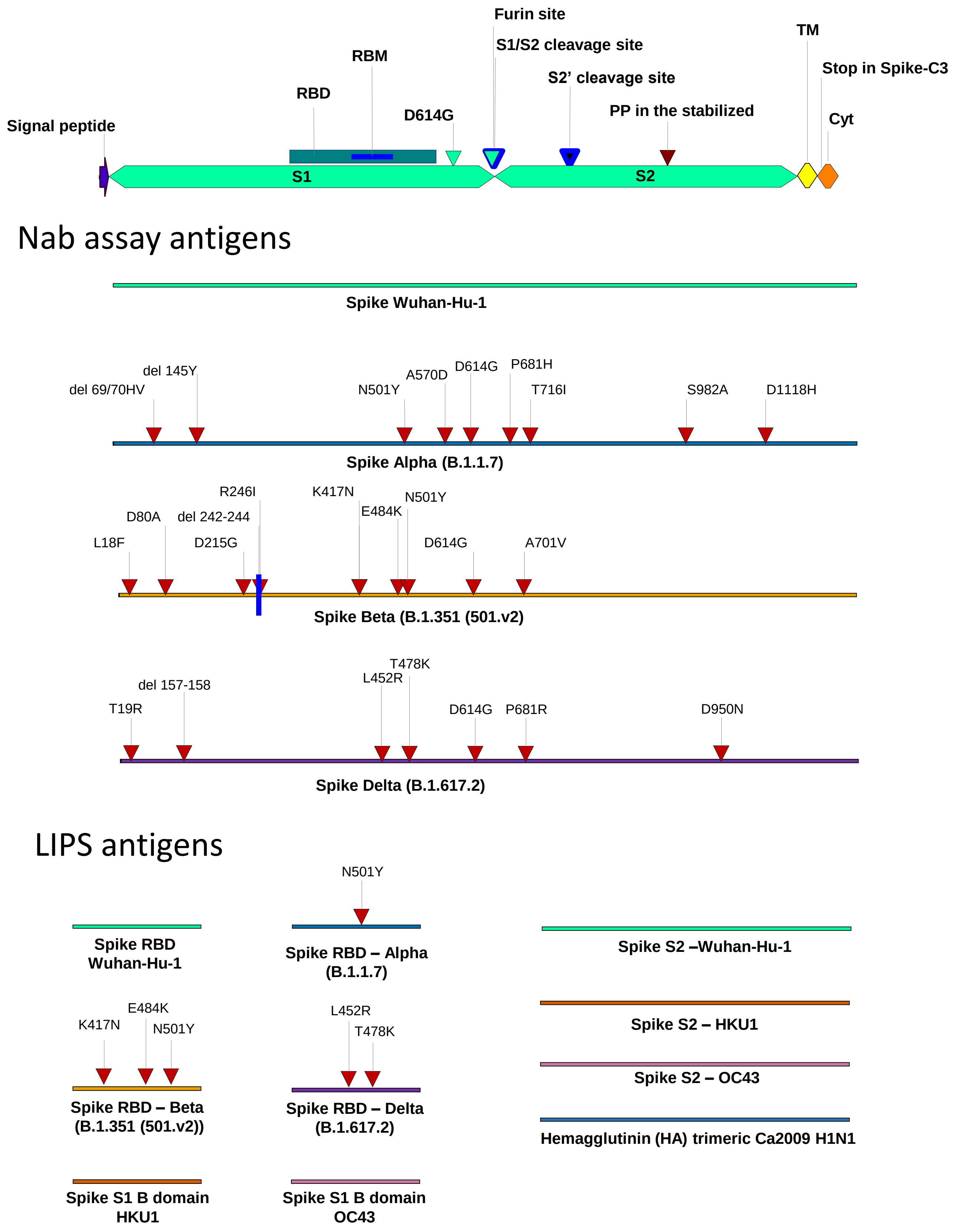

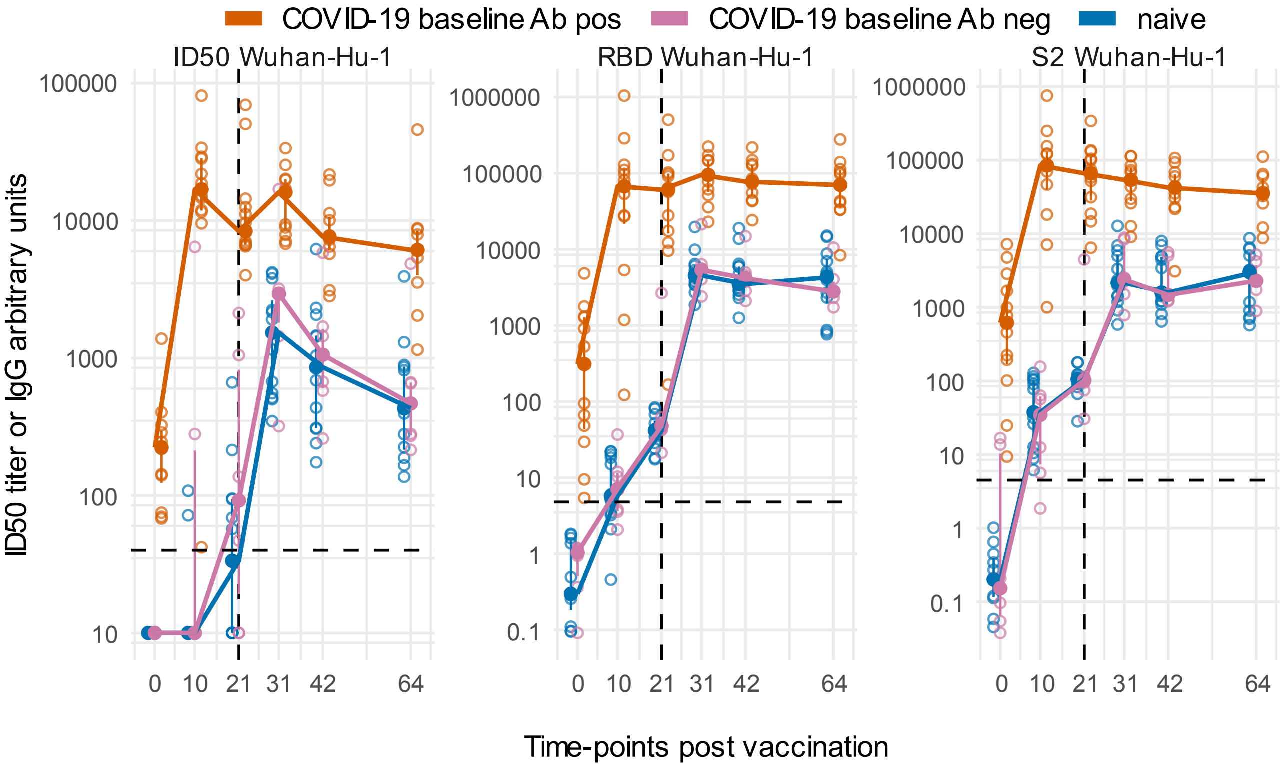

Antibody responses against Wuhan-Hu-1 spike antigens in BNT162b2 vaccinees stratified by SARS-CoV-2 Ab status at baseline Lineplots show the temporal profile post BNT162b2 vaccination of ID50 titers or IgG arbitrary units against the Spike RBD or S2 domains. Vaccinees are stratified according to previous infection with SARS-CoV-2 into naïve or with previous COVID-19 either without or with SARS-CoV-2 neutralizing and RBD antibodies at baseline before vaccination (COVID-19 baseline Ab neg or COVID-19 baseline Ab pos, respectively). Filled circles with error bars correspond to median ± IQR at the indicated timepoints. Empty circles correspond to individual subject values. Horizontal dashed lines indicate the respective assay threshold for positivity. The vertical dashed line indicates the second vaccine jab timepoint.

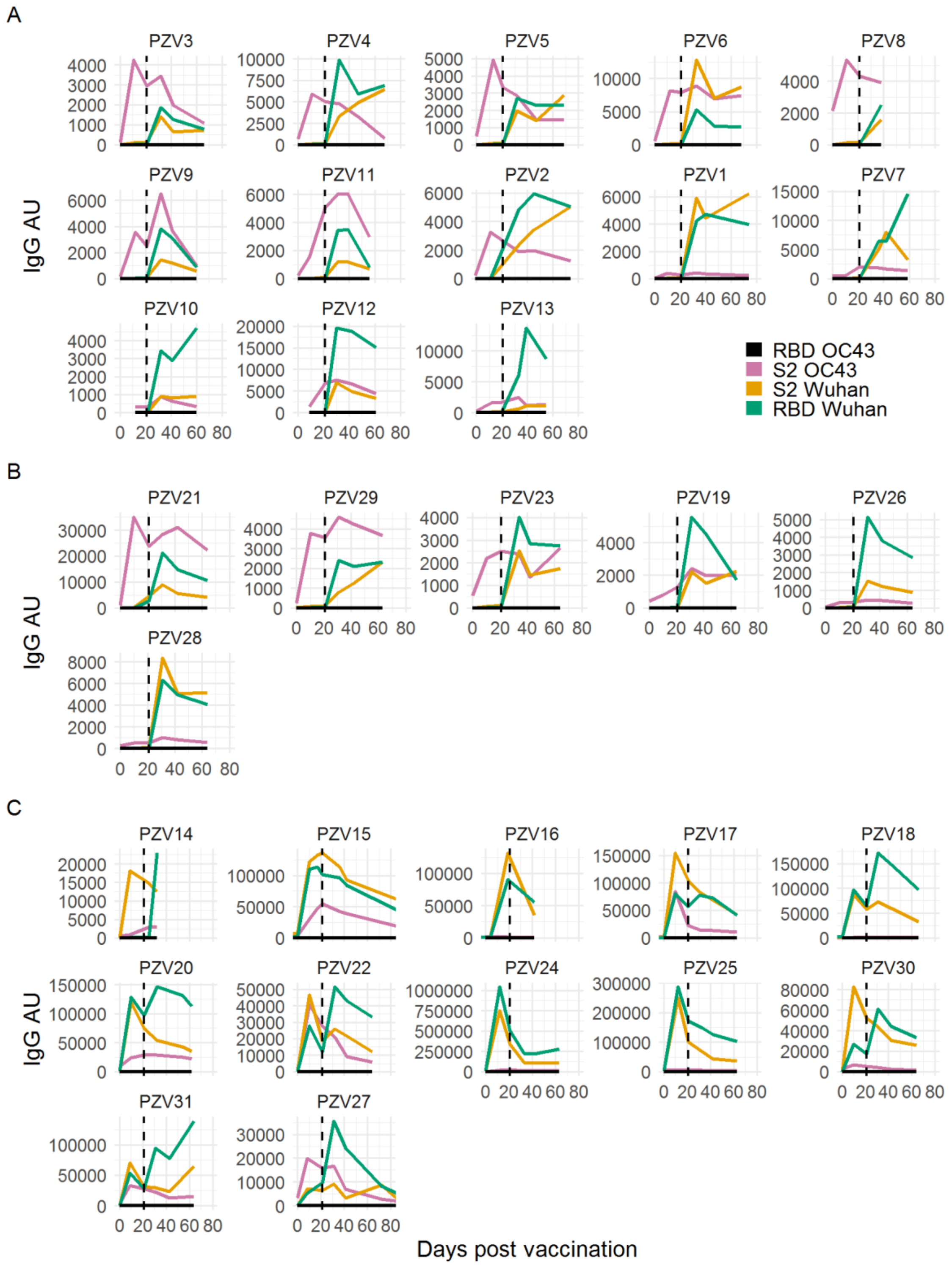

The early boost of seasonal betacoronavirus OC43 antibodies induced by BNT162b2-Comirnaty vaccination is directed against the spike S2 subunit but not the Spike S1 B domain. Lineplots of IgG arbitrary units (AU) against SARS-CoV-2 Wuhan-Hu-1 Spike S 1 RBD, Wuhan-Hu-1 spike S2 subunit, OC43 Spike S1 B domain, and OC43 spike S2 subunit at sequential time-points after vaccination. Vaccinees are stratified as: subjects naïve for SARS-CoV-2 infection (Panel A, n=13), subjects with prior confirmed COVID-19 presenting at vaccination either without Wuhan-Hu-1 nAbs and RBD IgGs (Panel B, n=6) and with prior COVID-19 and SARS-CoV-2 antibodies at baseline (Panel C, n=12). The vertical dashed line indicates the 2nd vaccine jab.

Supplemental Figure S4

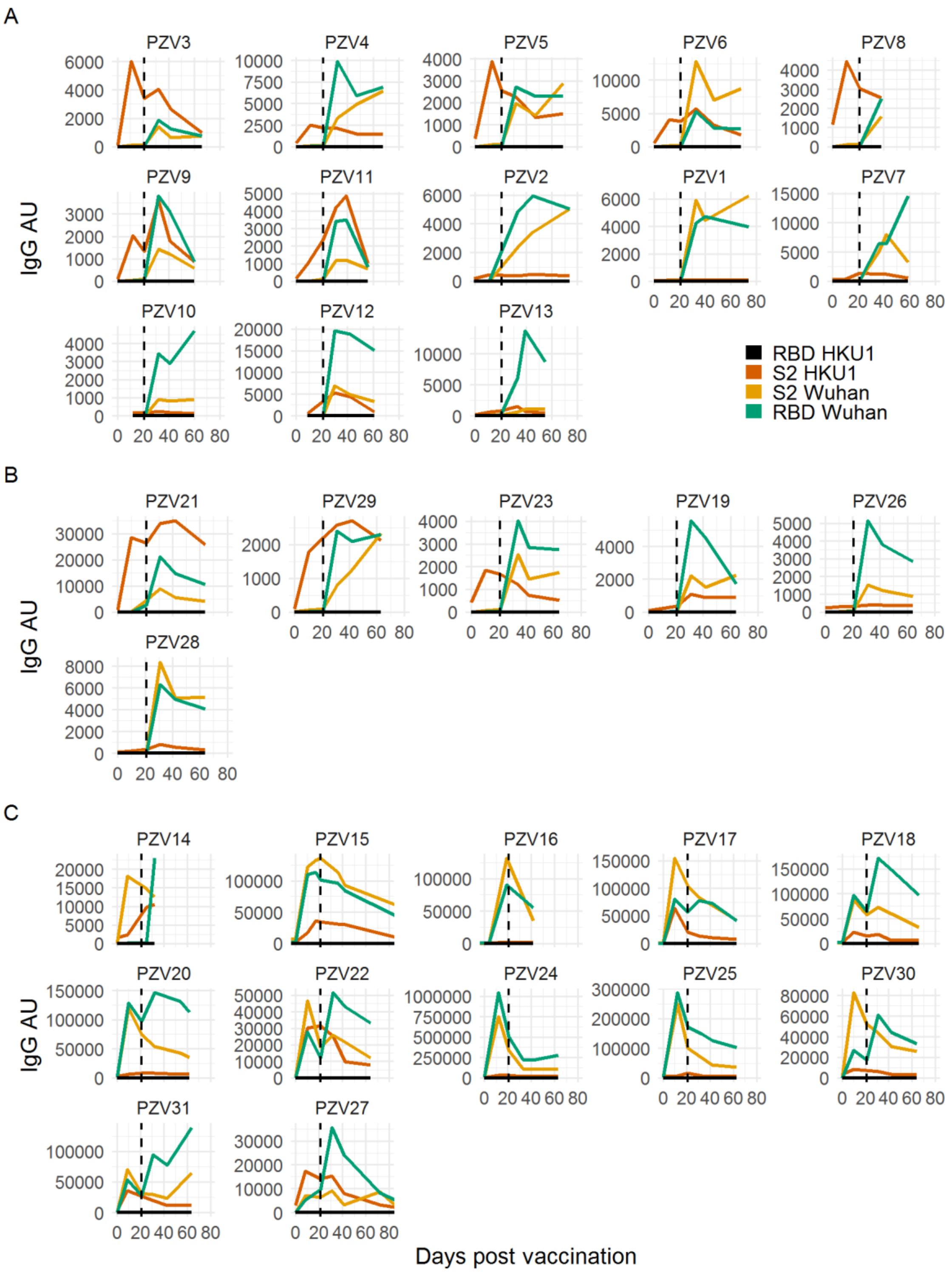

The early boost of seasonal betacoronavirus HKU1 antibodies induced by BNT162b2-Comirnaty vaccination is directed against the spike S2 subunit but not the Spike S1 B domain. Lineplots of IgG arbitrary units (AU) against SARS-CoV-2 Wuhan-Hu-1 Spike S 1 RBD, Wuhan-Hu-1 spike S2 subunit, HKU1 Spike S1 B domain, and HKU1 spike S2 subunit at sequential time-points after vaccination. Vaccinees are stratified as: subjects naïve for SARS-CoV-2 infection (Panel A, n=13), subjects with prior confirmed COVID-19 presenting at vaccination either without Wuhan-Hu-1 nAbs and RBD IgGs (Panel B, n=6) and with prior COVID-19 and SARS-CoV-2 antibodies at baseline (Panel C, n=12). The vertical dashed line indicates the 2nd vaccine jab.

A

Identities 28/108 (26%) Conservative substitutions 17/108 (16%)

```
OC43 S1 B domain     PDLPN-CNIEAWLNDKSVPSPLNWERKTFSNCNFNMSSLMSFIQADSFTCNNIDAAKIYG
                     P++ N C      N     S   W RK  SNC  + S L +     +F C   +  K+
SARS-CoV-2 S1 RBD    PNITNLCPFGEVFNATRFASVYAWNRKRISNCVADYSVLYNSASFSTFKCYGVSPTKLND

OC43 S1 B domain     MCFSSITIDKFAIPNRRKVDLQLGNLGYLQSSNYRIDTTATSCQLYYN   107
                     +CF+++  D F I       +  G  G +   NY++    T C + +N
SARS-CoV-2 S1 RBD    LCFTNVYADSFVIRGDEVRQIAPGQTGKIADYNYKLPDDFTGCVIAWN   119
```

B

Identities 220/513 (43%) Conservative substitutions 87/513 (17%)

```
OC43 S2 EC          IQIPSEFTIGNMEEFIQTSSPKVTIDCAAFVCGDYAACKLQLVEYGSFCDNINAILTEVN
                    I IP+ FTI    E +  S  K ++DC  ++CGD   C   L++YGSFC  +N  LT +
SARS-CoV-2 S2 EC    IAIPTNFTISVTTEILPVSMTKTSVDCTMYICGDSTECSNLLLQYGSFCTQLNRALTGIA

OC43 S2 EC          ELLDTTQLQVANSLMNGVTLSTKLKDGVNFNVDDINFAPVLGCLGSECSKASSRSAIEDL
                       D    +V  + +  +  +  +KD   FN   I   P         SK S RS IEDL
SARS-CoV-2 S2 EC    VEQDKNTQEVF-AQVKQIYKTPPIKDFGGFNFSQILPDP---------SKPSKRSFIEDL

OC43 S2 EC          LFDKVKLSDVGFVEAYNNCTGGAEIRDLICVQSYKGIKVLPPLLSENQISGYTLAATSAS
                    LF+KV L+D GF++ Y +C G    RDLIC Q + G+ VLPPLL++  I+ YT A  + +
SARS-CoV-2 S2 EC    LFNKVTLADAGFIKQYGDCLGDIAARDLICAQKFNGLTVLPPLLTDEMIAQYTSALLAGT

OC43 S2 EC          LFPPWTAAAG----VPFYLNVQYRINGLGVTMDVLSQNQKLIANAFNNALHAIQQGFDAT
                    +   WT  AG    +PF + + YR NG+GVT +VL +NQKLIAN FN+A+  IQ    +T
SARS-CoV-2 S2 EC    ITSGWTFGAGAALQIPFAMQMAYRFNGIGVTQNVLYENQKLIANQFNSAIGKIQDSLSST

OC43 S2 EC          NSALVKIQAVVNANSEALNNLLQQLSNRFGAISASLQEILSRLDALEAEAQIDRLINGRL
                     SAL K+Q VVN N++ALN L++QLS+ FGAIS+ L +ILSRLD +EAE QIDRLI GRL
SARS-CoV-2 S2 EC    ASALGKLQDVVNQNAQALNTLVKQLSSNFGAISSVLNDILSRLDKVEAEVQIDRLITGRL

OC43 S2 EC          TALNAYVSQQLSDSTLVKFSAAQAMEKVNECVKSQSSRINFCGNGNHIISLVQNAPYGLY
                     +L  YV+QQL  +  ++ SA  A  K++ECV  QS R++FCG G H++S  Q+AP+G+
SARS-CoV-2 S2 EC    QSLQTYVTQQLIRAAEIRASANLAATKMSECVLGQSKRVDFCGKGYHLMSFPQSAPHGVV

OC43 S2 EC          FIHFNYVPTKYVTAKVSPGLCIAGNRGIAPKSGYFVNVNNTWMYTGSGYYYPEPITENNV
                    F+H  YVP +      +P +C  G +   P+ G FV+    W  T   +Y P+ IT +N
SARS-CoV-2 S2 EC    FLHVTYVPAQEKNFTTAPAICHDG-KAHFPREGVFVSNGTHWFVTQRNFYEPQIITTDNT

OC43 S2 EC          VVMSTCAVNYTKAPYVMLNTSIPNLPDFKEELDQWFKNQTSVAPDLS-LDYINVTFLDLQ
                     V   C V        + +   P L  FKEELD++FKN TS   DL  +  IN + +++Q
SARS-CoV-2 S2 EC    FVSGNCDVVIGIVNNTVYDPLQPELDSFKEELDKYFKNHTSPDVDLGDISGINASVVNIQ

OC43 S2 EC          VEMNRLQEAIKVLNHSYINLKDIGTYEYYVKWP   510
                     E++RL E  K LN S I+L+++G YE Y+KWP
SARS-CoV-2 S2 EC    KEIDRLNEVAKNLNESLIDLQELGKYEQYIKWP   528
```

Spike protein amino acid sequence homology between SARS-CoV-2 and the OC43 seasonal betacoronavirus. Number of identical or conserved amino acid residues and sequence alignment of the SARS-CoV-2 spike S1 RBD and OC43 Spike S1 B domain (panel A). Number of identical or conserved amino acid residues and sequence alignment of the SARS-CoV-2 and OC43 Spike S2 subunits (panel B).

A

Identities 25/99 (25%) Conservative substitutions 13/99 (13%)

```
HKU1 S1 B domain     CDIDKWLNNFNVPSPLNWERKIFSNCNFNLSTLLRLVHTDSFSCNNFDESKIYGSCFKSI
                     C   +  N     S   W RK  SNC  + S L       +F C     +K+   CF ++
SARS-CoV-2 S1 RBD    CPFGEVFNATRFASVYAWNRKRISNCVADYSVLYNSASFSTFKCYGVSPTKLNDLCFTNV

HKU1 S1 B domain     VLDKFAIPNSRRSDLQLGSSGFLQSSNYKIDTTSSSCQL  104
                       D F I       +  G +G +   NYK+    + C +
SARS-CoV-2 S1 RBD    YADSFVIRGDEVRQIAPGQTGKIADYNYKLPDDFTGCVI  116
```

B

Identities 211/514 (41%) Conservative substitutions 96/514 (19%)

```
HKU1 S2 EC          IKIPTNFTIVGQEEFIQTNSPKVTIDCSLFVCSNYAACHDLLSEYGTFCDNINSILDEVN
                     I IPTNFTI    E +  +  K ++DC++++C +   C +LL +YG+FC  +N  L  +
SARS-CoV-2 S2 EC     IAIPTNFTISVTTEILPVSMTKTSVDCTMYICGDSTECSNLLLQYGSFCTQLNRALTGIA

HKU1 S2 EC          GLLDTTQLHVADTLMQGVTLSSNLNTNLHFDVDNINFKSLVGCLGPHCGSSSRSFFEDLL
                        D     V   + Q         T    D    NF  +   L      S RSF EDLL
SARS-CoV-2 S2 EC     VEQDKNTQEVFAQVKQ------IYKTPPIKDFGGFNFSQI---LPDPSKPSKRSFIEDLL

HKU1 S2 EC          FDKVKLSDVGFVEAYNNCTGGSEIRDLLCVQSFNGIKVLPPILSESQISGYTTAATVAAM
                     F+KV L+D GF++ Y +C G    RDL+C Q FNG+ VLPP+L++  I+ YT+A     +
SARS-CoV-2 S2 EC     FNKVTLADAGFIKQYGDCLGDIAARDLICAQKFNGLTVLPPLLTDEMIAQYTSALLAGTI

HKU1 S2 EC          FPPWSAAAG----IPFSLNVQYRINGLGVTMDVLNKNQKLIATAFNNALLSIQNGFSATN
                        W+  AG    IPF++ + YR NG+GVT +VL +NQKLIA  FN+A+  IQ+  S+T
SARS-CoV-2 S2 EC     TSGWTFGAGAALQIPFAMQMAYRFNGIGVTQNVLYENQKLIANQFNSAIGKIQDSLSSTA

HKU1 S2 EC          SALAKIQSVVNSNAQALNSLLQQLFNKFGAISSSLQEILSRLDALEAQVQIDRLINGRLT
                     SAL K+Q VVN NAQALN+L++QL + FGAISS L +ILSRLD +EA+VQIDRLI GRL
SARS-CoV-2 S2 EC     SALGKLQDVVNQNAQALNTLVKQLSSNFGAISSVLNDILSRLDKVEAEVQIDRLITGRLQ

HKU1 S2 EC          ALNAYVSQQLSDISLVKFGAALAMEKVNECVKSQSPRINFCGNGNHILSLVQNAPYGLLF
                     +L  YV+QQL   + ++  A LA  K++ECV  QS R++FCG G H++S  Q+AP+G++F
SARS-CoV-2 S2 EC     SLQTYVTQQLIRAAEIRASANLAATKMSECVLGQSKRVDFCGKGYHLMSFPQSAPHGVVF

HKU1 S2 EC          MHFSYKPISFKTVLVSPGLCISGDVGIAPKQGYFIKHNDHWMFTGSSYYYPEPISDKNVV
                     +H +Y P   K    +P +C  G     P++G F+ +  HW  T   ++Y P+ I+  N
SARS-CoV-2 S2 EC     LHVTYVPAQEKNFTTAPAICHDGKAHF-PREGVFVSNGTHWFVTQRNFYEPQIITTDNTF

HKU1 S2 EC          FMNTCSVNFTKAPLVYLNHSVPKLSDFESELSHWFKNQTSIAPNLTL-NLHTINATFLDL
                         C V          +   P+L  F+ EL  +FKN TS  P++ L ++  INA+ +++
SARS-CoV-2 S2 EC     VSGNCDVVIGIVNNTVYDPLQPELDSFKEELDKYFKNHTS--PDVDLGDISGINASVVNI

HKU1 S2 EC          YYEMNLIQESIKSLNNSYINLKDIGTYEMYVKWP  511
                       E++ + E  K+LN S I+L+++G YE Y+KWP
SARS-CoV-2 S2 EC     QKEIDRLNEVAKNLNESLIDLQELGKYEQYIKWP  528
```

Spike protein amino acid sequence homology between SARS-CoV-2 and the HKU1 seasonal betacoronavirus. Number of identical or conserved amino acid residues and sequence alignment of the SARS-CoV-2 spike S1 RBD and HKU1 Spike S1 B domain (panel A). Number of identical or conserved amino acid residues and sequence alignment of the SARS-CoV-2 and HKU1 Spike S2 subunits (panel B).

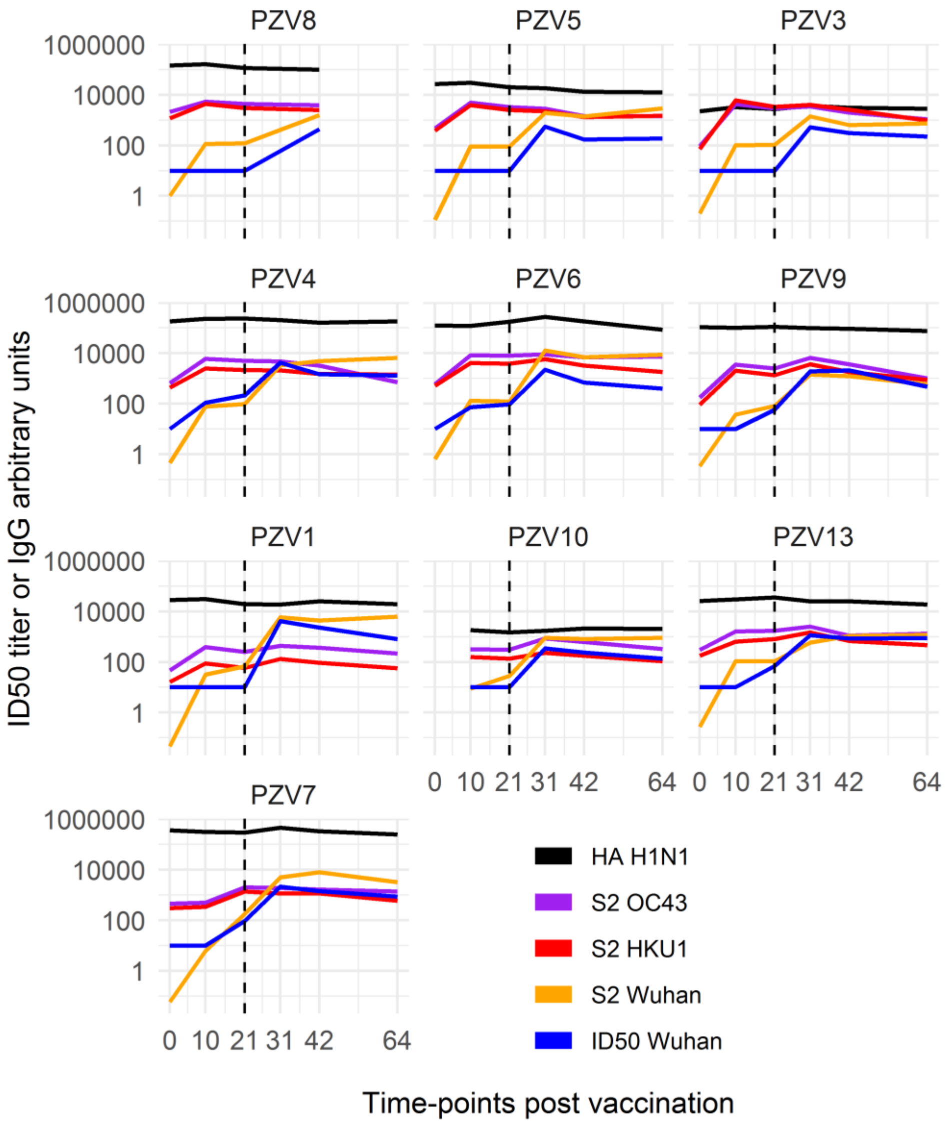

The antibody response to the pandemic H1N1 flu virus HA antigen is not affected by vaccination. The lineplots show the temporal profile post BNT162b2 vaccination of antibody levels in binding or neutralization assays in a selection of SARS-CoV-2 naïve BNT162b2 vaccinees. HA antibodies show modest fluctuation over time that are not synchronous with hose against betacoronaviruses' antigens. The dashed line indicates the second BNT162b2 jab timepoints.

Supplemental figure S8

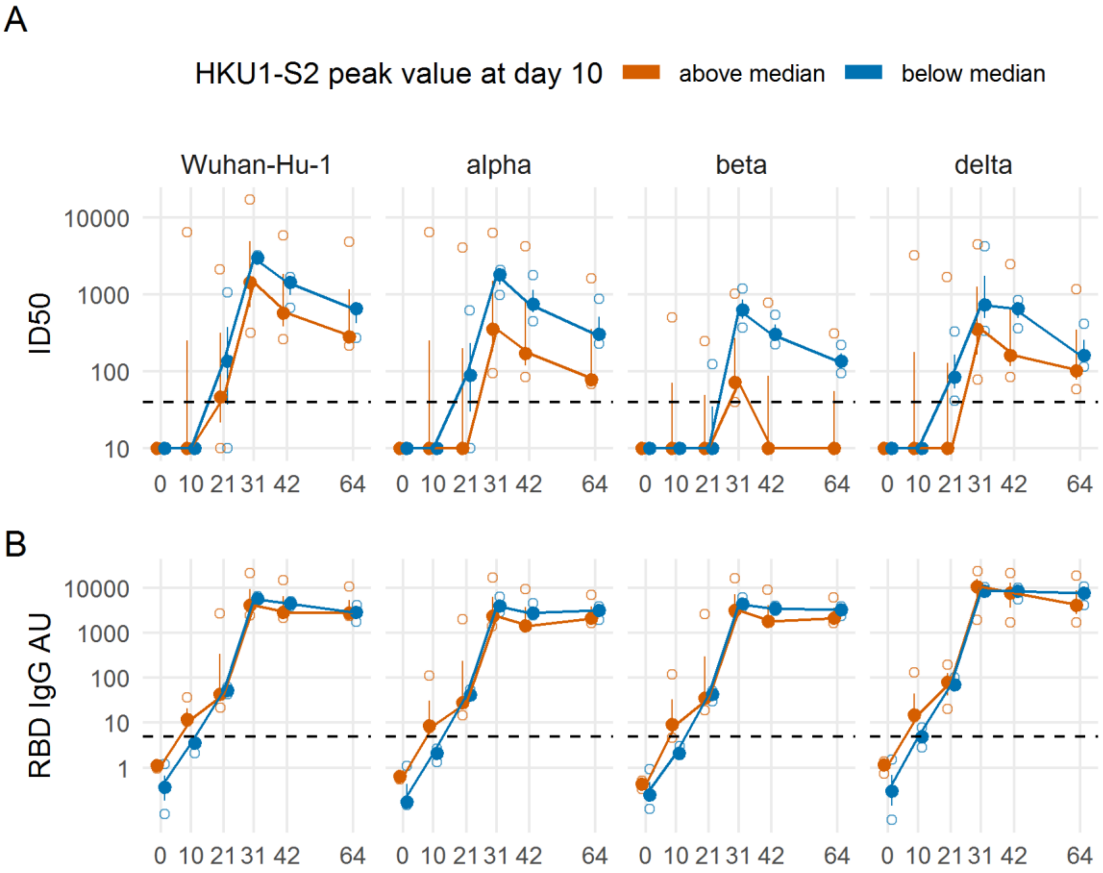

In vaccinees with previous COVID-19 and SARS-CoV-2 antibody negative at baseline, an early boost of HKU1 IgGs post-vaccination is associated with a trend towards a more rapid decrease of Nabs titers but not RBD IgG binding antibodies The lineplots show the temporal profile of antibody levels in neutralizing (upper panels) or binding (lower panels) antibody assays using antigens from the indicated SARS-CoV-2 variants. Subjects were stratified according to HKU1 S2 spike IgG levels above or below the median at day 10 post vaccination. The observed difference between the two strata did not reach statistical significance (two-way repeated measures ANOVA p adjusted = ns).

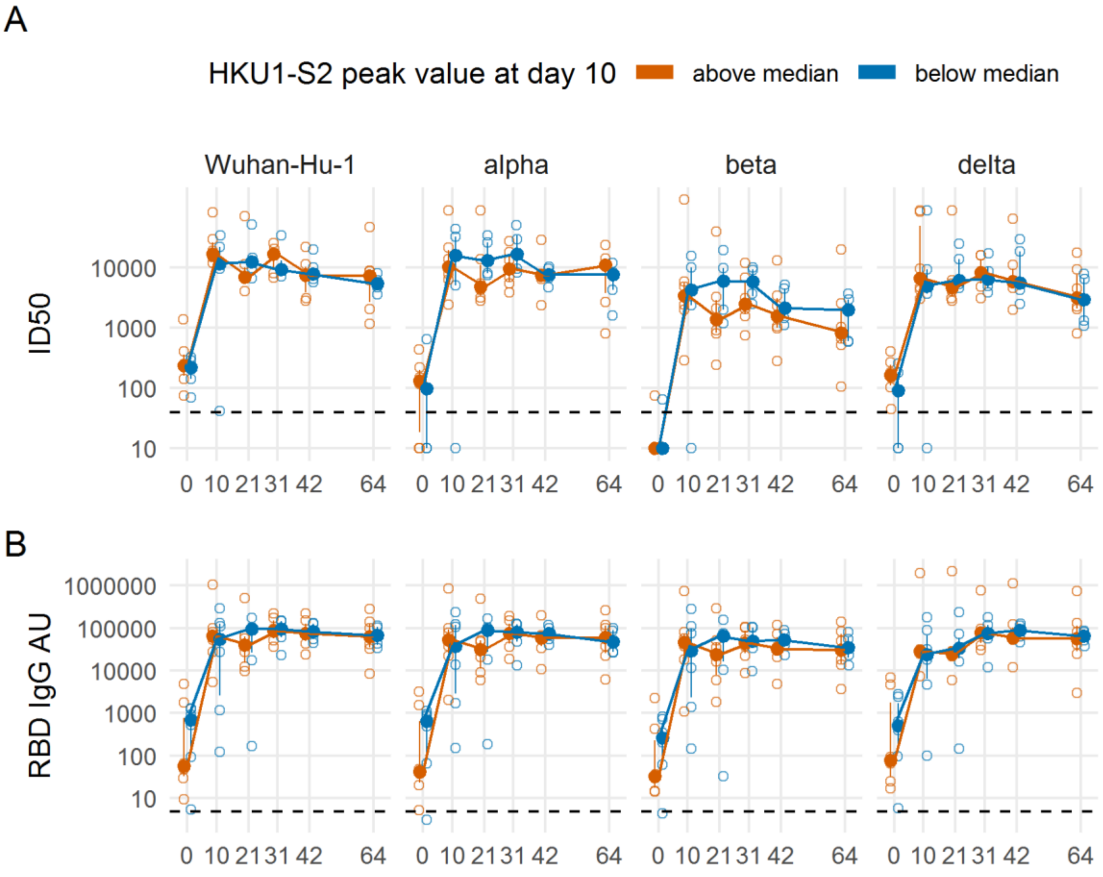

In vaccinees with previous COVID-19 and SARS-CoV-2 antibody positive at baseline, higher early IgG to the HKU1 S2 spike post BNT162b2 vaccination are not associated with later Nabs titers or RBD IgG binding antibodies The lineplots show the temporal profile of antibody levels in neutralizing (upper panels) or binding (lower panels) antibody assays using antigens from the indicated SARS-CoV-2 variants. Subjects were stratified according to HKU1 S2 spike IgG levels above or below the median at day 10 post vaccination. No significant differences between the two strata were observed in a two-way repeated measures ANOVA.
